# Clinical phenotypes of ischaemic stroke patients and early cardiovascular complications: a latent class analysis from the Perugia Stroke Registry

**DOI:** 10.1007/s11739-026-04420-z

**Published:** 2026-06-10

**Authors:** Andrea Galeazzo Rigutini, Valeria Caso, Tommaso Bucci, Maria Giulia Mosconi, Virginia Cancelloni, Andrea Alberti, Michele Venti, Ilaria Leone de Magistris, Alessandro Bufi, Chiara Urbini, Allegra Gaboni, Michele Marvardi, Carmen Rapuano, Wenhui Li, Cecilia Becattini, Gregory Y. H. Lip, Maurizio Paciaroni

**Affiliations:** 1 Internal, Vascular and Emergency Medicine – Stroke Unit, University of Perugia, Perugia, Italy; 2 Liverpool Centre for Cardiovascular Science at University of Liverpool, Liverpool John Moores University and Liverpool Heart & Chest Hospital, Liverpool, UK; 3 Neurology-Stroke Unit, Saronno Hospital-ASST Valle Olona, Saronno, Italy; 4 Emergency Department, Santa Maria Misericordia Hospital-Perugia, Perugia, Italy; 5 Operational Unit of Emergency Medicine and Urgent Care, Santa Maria Delle Grazie Hospital, Local Health Authority Naples, 2 North, Pozzuoli, Italy; 6 Department of Computer Science, Faculty of Environment, Science and Economy, University of Exeter, Exeter, UK; 7 Department of Clinical Medicine, Aalborg University, Aalborg, Denmark; 8 Department of Neurosciences and Rehabilitation Azienda Ospedaliero, Universitaria Di Ferrara, University of Ferrara, Arcispedale Sant’Anna, Via Aldo Moro 8, 44124 Cona Ferrara, Italy

**Keywords:** Ischaemic stroke, Embolic stroke of undetermined source, Latent class analysis, Phenotype

## Abstract

The heterogeneous pathophysiology of ischaemic stroke (IS) complicates risk stratification and therapeutic decision-making. Among aetiological subtypes, embolic strokes of undetermined source (ESUS) remain particularly challenging in terms of aetiology, management, and prognosis. The aim of this study was to identify distinct clinical phenotypes of IS with a specific emphasis on delineating the clinical landscape of ESUS. We analysed 1,533 patients with acute IS enrolled in the Perugia Stroke Registry (2005–2019). Latent class analysis (LCA) was used to identify clinical phenotypes based on demographics, vascular risk factors, stroke severity, and aetiology. A secondary LCA was performed among ESUS patients to explore sub-phenotypic heterogeneity. The primary composite outcome included all-cause death, cardiac events, and venous thromboembolism during hospital stay. Secondary outcomes included individual components of the primary outcome, haemorrhagic transformation (HT), and poor functional recovery (defined as mRS worsening from 0–2 to 3–6). Three phenotypes emerged using LCA, as follows: (i) non-cardioembolic (*n* = 761), predominantly male, high cardiovascular burden, moderate severity; (ii) cardioembolic (*n* = 557), older, mostly female, high prevalence of atrial fibrillation, more severe strokes; and (iii) ESUS (*n* = 215), younger, fewer comorbidities, heterogeneous severity. The cardioembolic class showed increased risk of the composite outcome (OR 2.18; 95% CI 1.40–3.50), all-cause death (OR 1.73; 95% CI 1.04–3.01), cardiac events (OR 2.62; 95% CI 1.42–5.34), HT (OR 1.65; 95% CI 0.99–2.88), and a trend towards poor functional recovery (OR 1.32; 95% CI 0.92–1.91), compared to ESUS. Among ESUS, three sub-phenotypes were identified: ‘Middle-age Dysmetabolic’, ‘Cardiovascular Elderly’, and ‘Low cardiovascular Young’. Compared to low cardiovascular young, both middle-age dysmetabolic and cardiovascular elderly phenotypes were associated with increased risk of the composite outcome (OR 1.52; 95% CI 1.18–2.83 and OR 1.56; 95% CI 1.21–2.77, respectively). Acute IS is clinically heterogeneous; ESUS differs from cardioembolic strokes in management and short-term outcomes. The three ESUS sub-phenotypes highlight the need for more personalised strategies in diagnosis and secondary prevention.

**Supplementary Information:**

The online version contains supplementary material available at https://doi.org/10.1007/s11739-026-04420-z.

## Introduction

Ischaemic stroke (IS) remains a leading cause of mortality and long-term disability worldwide, representing a major contributor to global disease burden [1]. Despite improvements in acute management, clinical outcomes remain highly variable, reflecting the complex and heterogeneous nature of the IS [2].

While conventional aetiological classifications, including the Trial of Org 10,172 in Acute Stroke Treatment (TOAST) criteria [3], provide diagnostic guidance, they do not fully account for the clinical complexity of IS. In particular, although the cardioembolic and atherothrombotic stroke subtypes are well characterised [4–6], embolic strokes of undetermined source (ESUS) remains a diagnostic and therapeutic challenge, with unresolved uncertainties in aetiology, treatment, and risk stratification [7–12].

This lack of phenotypic clarity hampers personalised care and risk prediction. Refined phenotyping may improve stratification, guide treatment, and anticipate systemic complications, particularly in the early post-stroke phase when neuronal injury triggers systemic inflammation and increases cardiovascular risk [13–15].

Nonetheless, evidence on how distinct clinical profiles relate to acute treatment patterns, early cardiovascular complications, and functional recovery remains limited. To address this knowledge gap, we first performed a latent class analysis (LCA) to identify distinct clinical phenotypes of IS with a specific emphasis on delineating the clinical landscape of ESUS. Second, we then examined differences across these phenotypes in terms of acute treatment strategies and early clinical outcomes, including cardiovascular complications, venous thromboembolism, haemorrhagic transformation, and functional recovery at hospital discharge.

## Methods

### Study design and population

We analysed data from the Perugia Stroke Registry, a retrospective hospital-based registry including consecutive patients admitted to the Stroke Unit of Santa Maria della Misericordia University Hospital (Perugia, Italy) between March 2005 and September 2019. Inclusion did not influence patient management. Patients were eligible if they presented with acute focal neurological deficits and had a radiologically confirmed diagnosis of ischaemic or haemorrhagic stroke. Patients with subarachnoid haemorrhage, venous sinus thrombosis, or stroke mimics were excluded. Further details on study design and imaging protocols are provided in the Supplementary Methods 1.

### Data collection

Demographic and clinical characteristics, vascular risk factors, comorbidities, pre- and in-hospital treatments, and in-hospital complications were systematically recorded. The presence of HT was defined according to the European Cooperative Acute Stroke Study (ECASS) criteria [16], including any haemorrhagic infarction or parenchymal haemorrhage. Symptomatic intracerebral haemorrhage (sICH) was defined according to the ECASS II/III criteria, as the presence of a parenchymal haemorrhage on neuroimaging associated with a clinical deterioration of ≥ 4 points on the NIHSS or death [17]. Stroke severity was assessed using the National Institutes of Health Stroke Scale (NIHSS) at admission and discharge. Functional status was assessed using the modified Rankin Scale (mRS) at hospital admission and at discharge. Full diagnostic workup are detailed in Supplementary Methods 1.

### Latent class analysis

LCA was performed to identify unobserved patient subgroups based on demographic, vascular, and clinical characteristics. We constructed two models: a reduced model including age, sex, major vascular risk factors, atrial fibrillation, and NIHSS at admission; and a full model additionally including stroke aetiology (TOAST criteria: large artery atherosclerosis, small vessel occlusion, cardioembolic stroke, and ESUS). The optimal number of classes was determined using information criteria and clinical interpretability. Each patient was assigned to the class with the highest posterior probability. Further methodological details, including sensitivity analyses restricted to ESUS patients, are reported in **Supplementary Methods 2**.

### Outcomes

The primary outcome was the occurrence, during Stroke Unit stay, of a composite endpoint comprising all-cause of death, cardiac events (acute heart failure and acute coronary syndrome), and venous thromboembolic events (pulmonary embolism with or without deep vein thrombosis).

Secondary outcomes included the intrahospital risk of.

i) each component of the composite outcome assessed individually,
ii) HT and sICH,
iii) poor functional recovery.

For the purpose of this study, poor functional recovery was defined as a worsening of functional status from a mRS score of 0–2 (preserved functional independence) at admission to a score of 3–6 (significant disability or worse outcomes) at hospital discharge.

### Statistical analysis

Categorical variables were reported as frequencies and percentages and compared using the χ^2^ test or Fisher’s exact test, as appropriate. Continuous variables were expressed as mean ± standard deviation (SD) or median with interquartile range (IQR) and compared using the student’s t-test or Mann–Whitney U test, depending on data distribution.

We calculated the in-hospital incidence of a composite outcome defined as the occurrence of all-cause mortality, cardiovascular complications (acute myocardial infarction or heart failure), or venous thromboembolic events (pulmonary embolism or deep vein thrombosis). The incidence of each individual component of the composite outcome was also assessed separately. In addition, we evaluated the incidence of HT and sICH. Incidence was expressed as the proportion of patients experiencing the event during Stroke Unit stay, reported with 95% confidence intervals (CIs), and compared across latent classes using the χ^2^ test.

Univariable logistic regression models were used to assess the association between latent classes and

i) acute stroke treatment strategies, including DAPT, IVT, and EVT,
ii) in-hospital clinical outcomes, including the composite endpoint, its individual components, HT, sICH and poor functional recovery.

All logistic models were unadjusted for additional covariates to avoid minimising the intrinsic clinical differences associated with each latent class. In all regression models, the ESUS class was used as the reference group, as the main focus of this study was to better characterise its clinical profile and early outcomes in comparison with non-cardioembolic stroke (including large artery atherosclerosis and small vessel occlusion) and cardioembolic stroke.

Forest plots were generated to visually represent odds ratios (ORs) and 95% CIs derived from the regression models. A Sankey diagram was constructed to illustrate the relationship between latent class membership, NIHHS at admission, and mRS at discharge, while a stratified bar plot was used to visualise the distribution of functional outcomes across latent classes and NIHSS categories.

All statistical analyses were performed using R software version 4.4.2 [18]. A two-sided P-value < 0.05 was considered statistically significant.

## Results

Among 2,131 patients with IS recorded in the registry, 1,533 individuals (72% of the entire cohort; mean age: 73 ± 12; female sex: 43%) had complete data on relevant clinical variables and were included in the LCA (Flow chart Supplementary Fig. 1). Patients included in the LCA tended to be younger (73 ± 12 vs. 74 ± 13, p-value 0.11), had a higher prevalence of AF (35% vs. 23%, p-value < 0.001), but had similar cardiovascular burden and stroke severity compared to those not included (Supplementary Table 1).

### LCA initial assessment

**Fig. 1:**
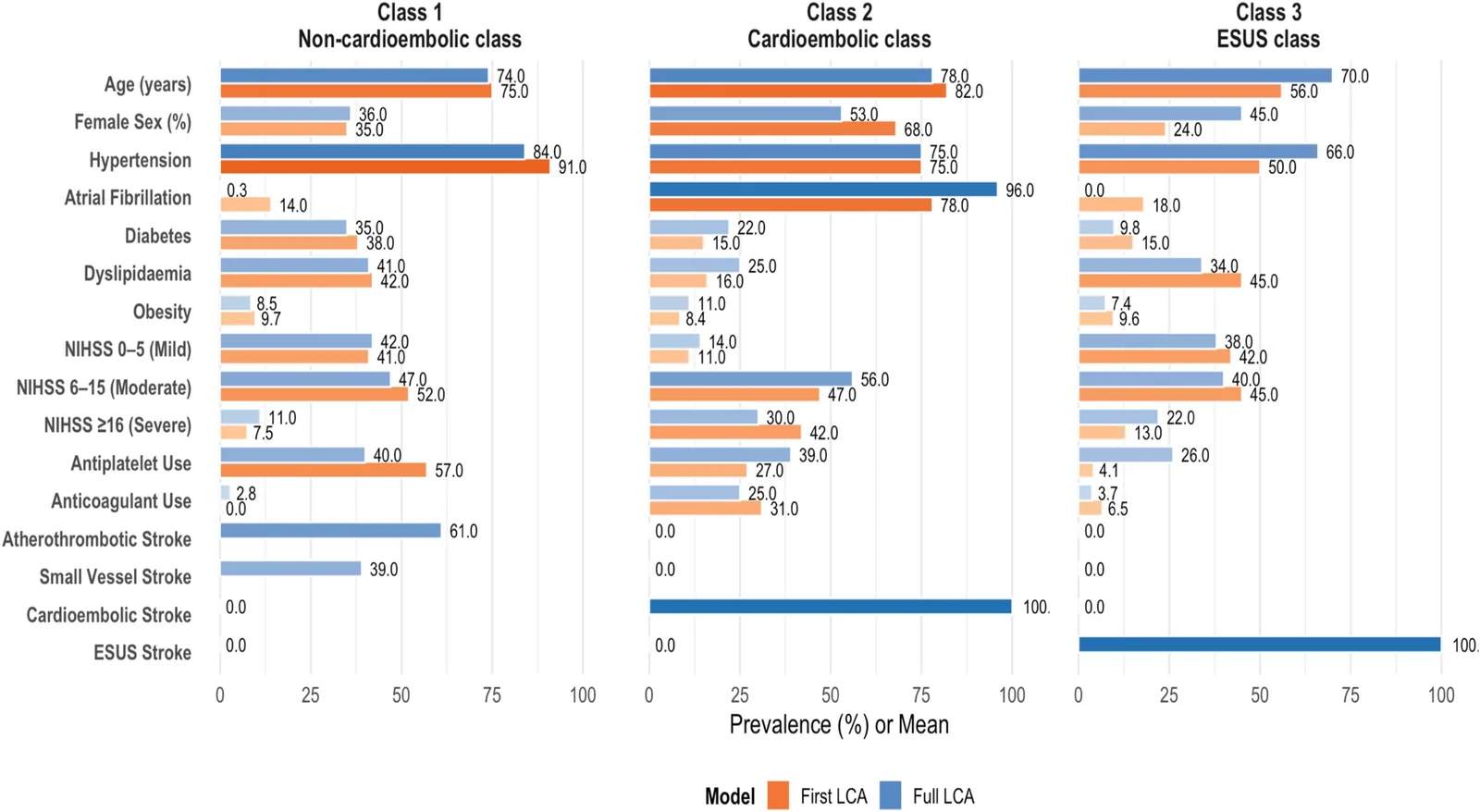
Comparison of class characteristics between first and full latent class models

When applying the first LCA model, distinct latent classes emerged. After taking into account of model fit indices, the three-class model was retained as the most robust (Fig. 1 and Supplementary Table 2, Supplementary Results 1). Class 1 (*n* = 763) was characterised by older age (median age 75 years, IQR 69–80), a high proportion of male sex (65%) and a substantial cardiovascular comorbidity burden (91% hypertension, 38% diabetes, 42% dyslipidaemia). This group predominantly experienced mild-to-moderate strokes and were frequently prescribed antiplatelet therapy (57%). Class 2 (*n* = 479) included predominantly elderly women (median age 82, IQR 77–86, female sex 68%) with a high prevalence of AF (78%) and a significant proportion presenting with severe strokes (42% NIHSS ≥ 16). This group demonstrated the highest rate of pre-admission oral anticoagulant use (31%). Class 3 (*n* = 291) represented younger cohort (median age 56, IQR 49–63), predominantly male (76%), with lower overall comorbidity burden (hypertension 50%, AF 18%, diabetes 15%, dyslipidaemia 42%). These patients typically experienced mild-to-moderate severity strokes and had minimal exposure to pre-stroke antithrombotic therapy.

When applying the second full LCA model, incorporating stroke aetiological information, a comparable class structure emerged. After taking into account of model fit indices, the three-class model was retained as the most robust: Class 1 (*n* = 761, “Non-cardioembolic class”), Class 2 (*n* = 557 (“Cardioembolic class”), and Class 3 (*n* = 215, “ESUS class”). Classes 1 and 2 remained largely consistent with the profiles identified in the original model, Class 3, while retaining its hallmark features—younger age, lower comorbidity burden, and a more heterogeneous stroke severity distribution—exhibited greater divergence (Fig. 1 and Table 1; Supplementary Results 1.). This group appeared to undergo a refinement process, suggesting that the model reallocated patients were potentially misclassified in the initial structure (Fig. 1 and Supplementary Table 2). A more comprehensive description of the main characteristics of each class derived from the full LCA model is provided in the Supplementary Results 1.

**Table 1:** Clinical characteristics across latent classes in ischaemic stroke patient (full model)

Characteristic	Class 1 *N* = 761^*1*^	Class 2 *N* = 557^*1*^	Class 3 *N* = 215^*1*^	p-value^*2*^
Age, years	74 (66, 81)	78 (71, 84)	70 (55, 79)	< 0.001
Female	273 (36%)	294 (53%)	97 (45%)	< 0.001
Hypertension	640 (84%)	415 (75%)	142 (66%)	< 0.001
Atrial fibrillation	2 (0.3%)	532 (96%)	0 (0%)	< 0.001
Diabetes	264 (35%)	120 (22%)	21 (9.8%)	< 0.001
Dyslipidaemia	313 (41%)	140 (25%)	73 (34%)	< 0.001
Obesity	65 (8.5%)	61 (11%)	16 (7.4%)	0.2
NIH baseline				
Mild (0–4)	282 (37%)	124 (22%)	79 (37%)	< 0.001
Moderate (5–15)	398 (52%)	267 (48%)	88 (41%)	
Severe (≥ 16)	81 (11%)	166 (30%)	48 (22%)	
Antiplatelet pre-hospital	304 (40%)	215 (39%)	56 (26%)	< 0.001
Anticoagulant therapy pre-hospital	21 (2.8%)	138 (25%)	8 (3.7%)	< 0.001
Atherothrombotic stroke	465 (61%)	0 (0%)	0 (0%)	< 0.001
Small vessels stroke	296 (39%)	0 (0%)	0 (0%)	< 0.001
Cardioembolic stroke	0 (0%)	557 (100%)	0 (0%)	< 0.001
ESUS stroke	0 (0%)	0 (0%)	215 (100%)	< 0.001

^*1*^ Median (Q1, Q3); n (%)

^*2*^ Kruskal–Wallis rank sum test; Pearson’s Chi-squared test

In this model, stroke aetiologies aligned well with their respective clusters, reinforcing the phenotypes identified by the first model and improving classification accuracy (Supplementary Fig. 2). Indeed, posterior probabilities from the full model indicated excellent certainty in class assignment (minimum: 0.988; 1 st quartile: 0.996; median: 0.998; 3rd quartile: 0.999). In contrast, the first model showed greater uncertainty, especially at the lower end of the range (minimum: 0.355; 1 st quartile: 0.706; median: 0.866).

Based on these findings, the full model including stroke aetiology was selected for final phenotype identification. The resulting classes were labelled according to the predominant clinical and vascular characteristics observed within each group.

### Acute treatments

DAPT was administrated in 19.1% of patients in the non-cardioembolic class, 7.5% in the cardioembolic class, and 11.2%% in the ESUS class. In the logistic regression analysis, patients in the non-cardioembolic class were significantly more likely to receive DAPT compared to those in the ESUS class (OR 1.67; 95% CI 1.05–2.67), whereas those in the cardioembolic class were significantly less likely to receive this treatment (OR 0.58; 95% CI 0.35–0.99).

IVT was most frequently administered in the ESUS class (17.7%), followed by the cardioembolic class (12.0%) and non-cardioembolic class (9.7%) classes. Logistic regression analysis confirmed that patients in the cardioembolic class (OR 0.59; 95% CI 0.38–0.91) and non-cardioembolic class (OR 0.54; 95% CI 0.35–0.83) classes had significantly lower odds of receiving IVT compared to those in the ESUS class.

Lastly, EVT was administered at similarly low rates across classes: 1.9% in the ESUS class, 1.3% in the non-cardioembolic class, and 1.1% in the cardioembolic class. No statistically significant associations were observed in logistic regression analysis for EVT (cardioembolic OR 0.50; 95% CI 0.14–1.79; non-cardioembolic (OR 0.21; 95% CI 0.02–2.24) compared to ESUS.

**Fig. 2:**
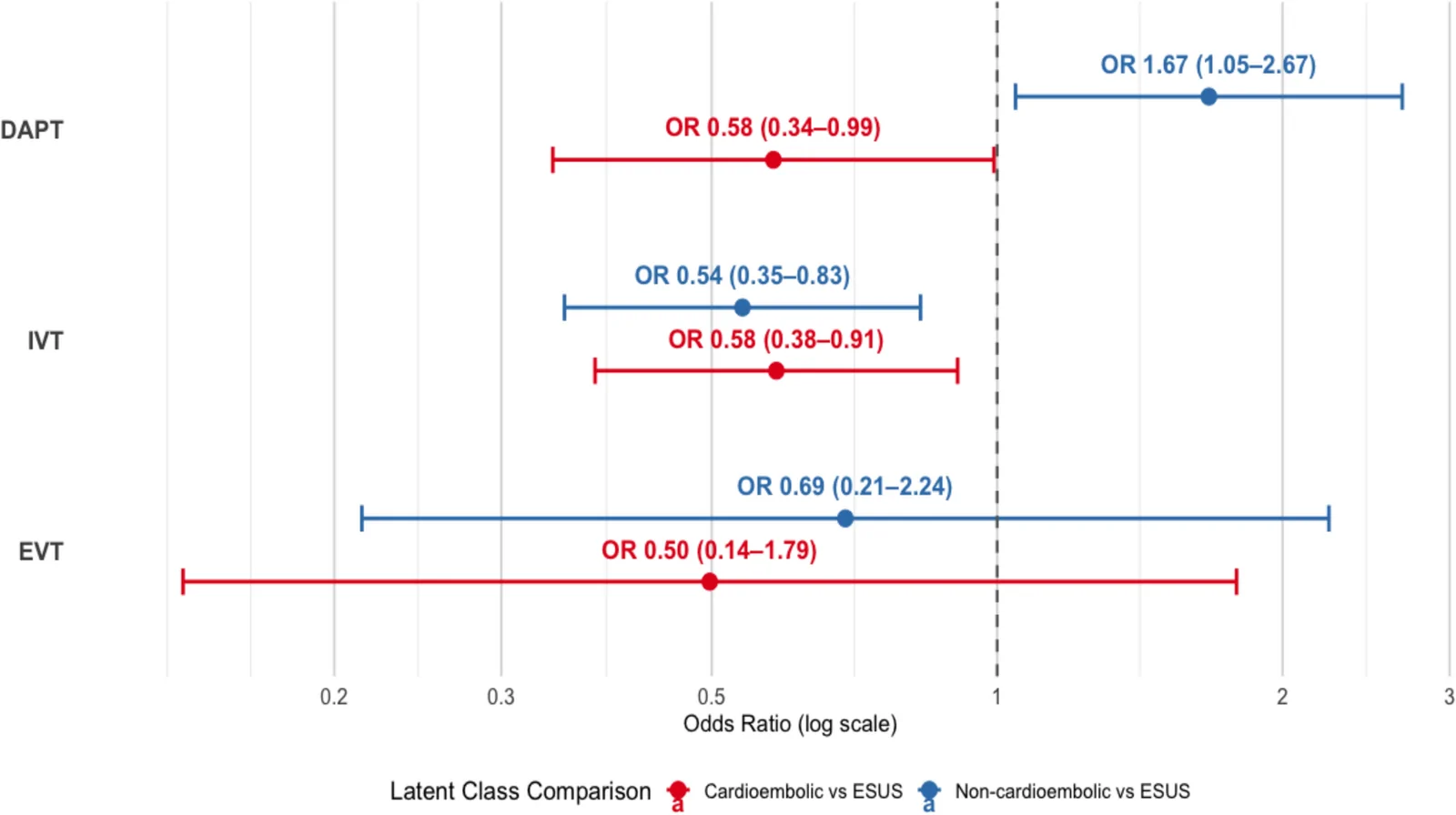
Forest plot about the association between latent classes and acute treatments at admission

Table 2 presents the distribution of acute treatment strategies across the latent classes, while Fig. 2 displays the results of the corresponding logistic regression analyses.

**Table 2:** Association between latent classes and acute treatment strategies at admission

Revascularisation types	Number of events (within class %)	Univariable analysis OR (95% CI)	p-value^*2*^
DAPT only			
Cardioembolic	42 (7.5%)	0.58 (0.35–0.99)	0.047
Non-cardioembolic	145 (19.1%)	1.67 (1.05–2.67)	0.032
ESUS	24 (11.2%)	Reference	
IVT only			
Cardioembolic	67 (12%)	0.59 (0.38–0.91)	0.017
Non-cardioembolic	74 (9.7%)	0.54 (0.35–0.83)	0.005
ESUS	38 (17.7%)	Reference	
EVT only			
Cardioembolic	6 (1.1%)	0.50 (0.14–1.79)	0.285
Non-cardioembolic	10 (1.3%)	0.21 (0.21–2.24)	0.538
ESUS	4 (1.9%)	Reference

### Risk of adverse events

The composite outcome was most frequent in the cardioembolic class, occurring in 23.2% of patients, followed by 12.1% in the ESUS class and 8.8% in the non-cardioembolic class.

A similar gradient was observed for all-cause death, with rates of 14.4% in the cardioembolic class, 8.8% in ESUS, and 5.4% in the non-cardioembolic class. Cardiac events occurred in 12.6% of cardioembolic class, compared with 5.1% in ESUS and 4.3% in the non-cardioembolic class.

Thromboembolic events were rare overall, with incidences of 2.2% in the cardioembolic class, 0.5% in ESUS, and 0.4% in the non-cardioembolic class (Table 3).

**Table 3:** Event rates and adjusted risk for in-hospital outcomes according to latent class

	Number of events	In-hospital incidence rate (95% CI)	Univariable analysis OR (95% CI)
Composite outcome			
Cardioembolic	129	23.2	2.18 (1.40–3.50)
Non-cardioembolic	67	8.8	0.69 (0.43–1.13)
ESUS	26	12.1	Reference
All-cause death			
Cardioembolic	80	14.4	1.73 (1.04–3.01)
Non-cardioembolic	41	5.4	0.59 (0.34–1.06)
ESUS	19	8.8	Reference
Cardiac events			
Cardioembolic	70	12.6	2.62 (1.42–5.34)
Non-cardioembolic	33	4.3	0.82 (0.42–1.73)
ESUS	11	5.1	Reference
Thromboembolic events			
Cardioembolic	12	2.2	4.65 (0.91–85.00)
Non-cardioembolic	3	0.4	0.83 (0.11–16.80)
ESUS	1	0.5	Reference
Haemorrhagic Transformation			
Cardioembolic	77	13.8	1.65 (0.99–2.88)
Non-cardioembolic	29	3.8	0.41 (0.23–0.77)
ESUS	19	5.1	Reference

In logistic regression analysis, the cardioembolic class was significantly associated with an increased risk of the composite outcome (OR 2.18; 95% CI, 1.40–3.50), all-cause death (OR 1.73; 95% CI, 1.04–3.01), and cardiac events (OR 2.62; 95% CI, 1.42–5.34) compared with the ESUS class.

**Fig. 3:**
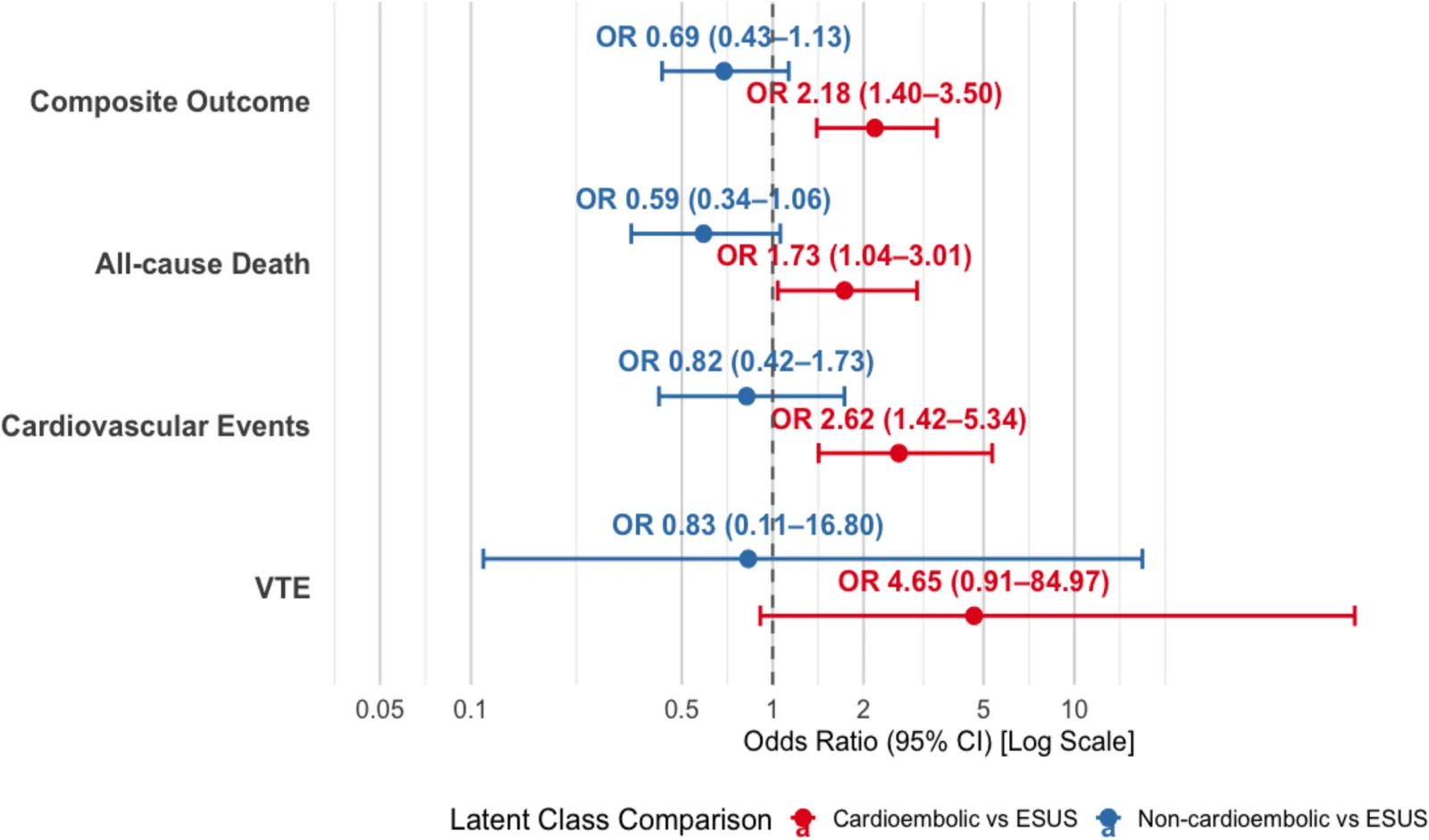
Forest plot showing association between latent classes and risk of in-hospital clinical outcomes

In contrast, the non-cardioembolic class showed a trend towards reduced risk of composite outcome (OR 0.69, 95% CI 0.43–1.13) and all-cause death (OR 0.59; 95% CI 0.34–1.06) compared to ESUS class. No associations were found for cardiac and venous thromboembolic events (Fig. 3).

### Haemorrhagic transformation and symptomatic intracerebral haemorrhage

**Fig. 4:**
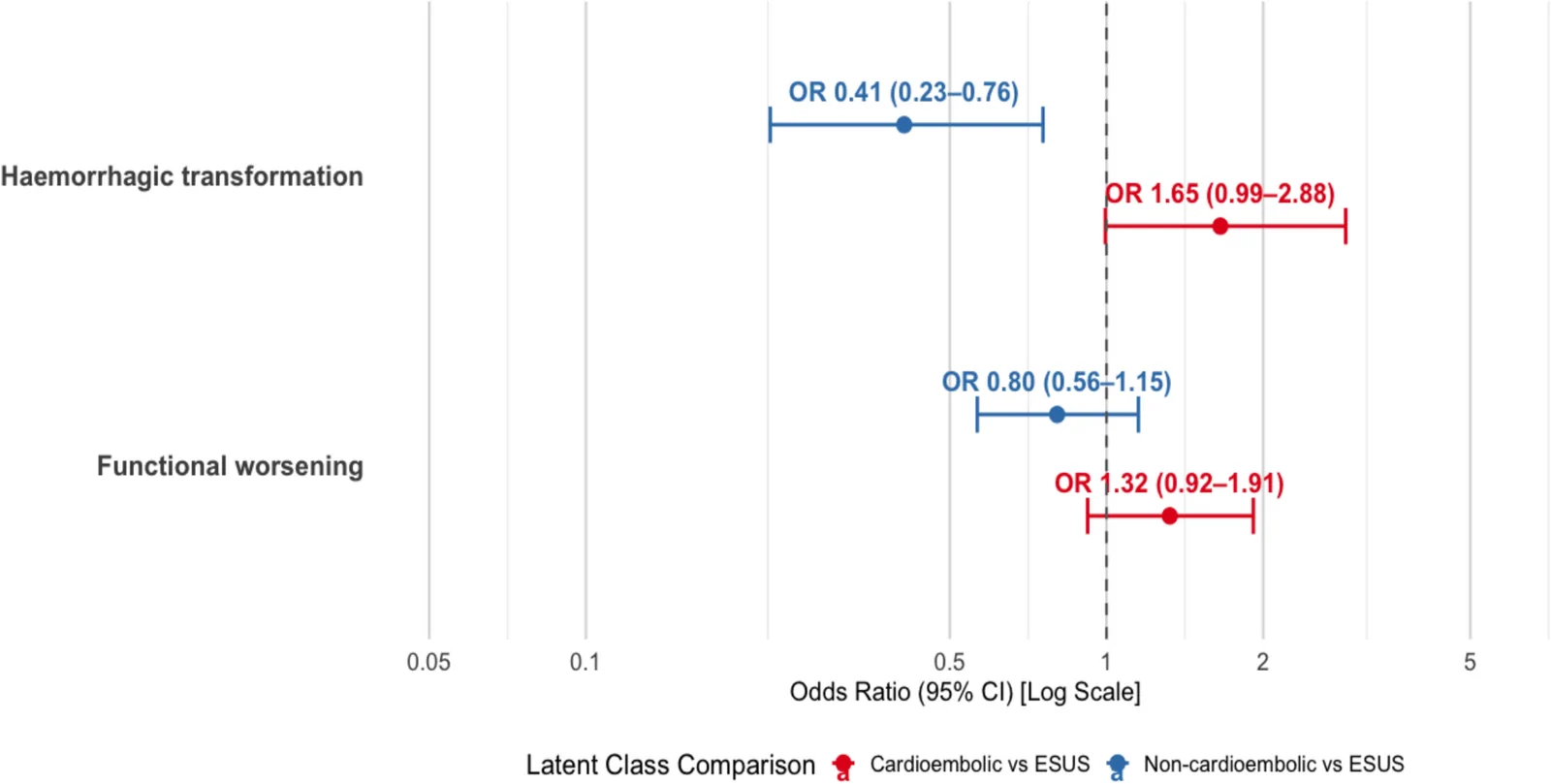
Forest plot about the association between latent classes and functional worsening or haemorrhagic transformation during hospitalisation

HT was reported in 13.8% of patients in the cardioembolic class, compared with 5.1% in ESUS and 3.8% in the non-cardioembolic class. In logistic regression analysis, the non-cardioembolic class was associated with a significantly lower risk of HT (OR 0.41; 95% CI, 0.23–0.77), while the cardioembolic class showed a higher risk (OR 1.65; 95% CI, 0.99–2.88) compared both to ESUS class (Fig. 4). Among patients with HT, sICH occurred in 11.1% of ESUS cases, 30.8% of non-cardioembolic cases, and 17.4% of cardioembolic cases. On multivariable logistic regression, the cardioembolic class was associated with an increased, though non-significant, risk of sICH (OR 1.68, 95% CI 0.40–11.54; *p* = 0.520), while the non-cardioembolic class demonstrated the highest, albeit non-significant, risk (OR 3.56, 95% CI 0.75–25.92; *p* = 0.140), compared with the ESUS class (Supplementary Table 3).

### Functional recovery according to latent classes

**Fig. 5:**
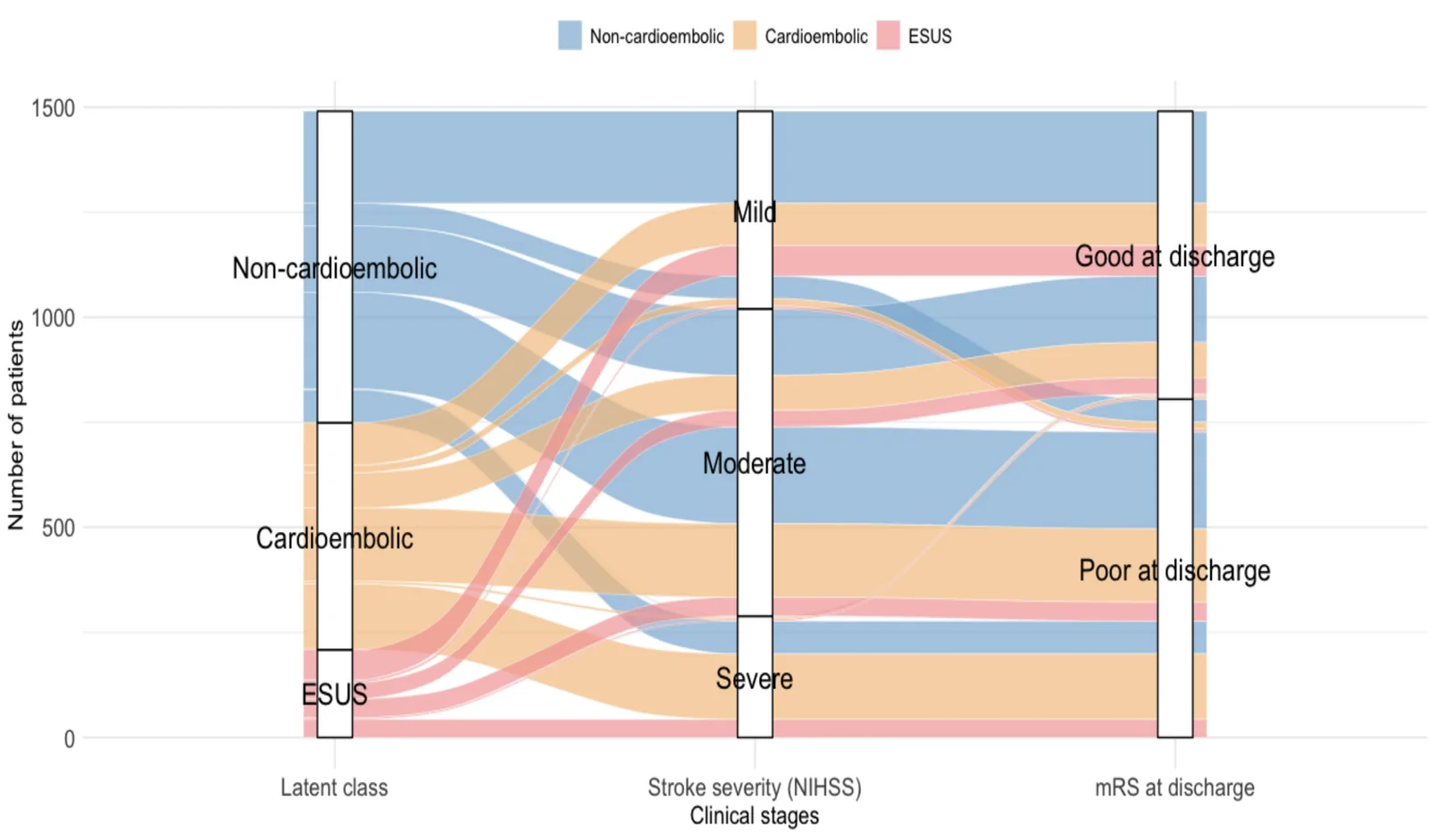
Latent class influence on functional recovery pathways during hospital stay

The Sankey diagram offers a comprehensive overview of the dynamic relationship between latent class, NIHSS at admission, and mRS at discharge (Fig. 5). Patients in the ESUS class with mild–moderate strokes frequently achieved favourable recovery, whereas those in the cardioembolic class more often presented with severe strokes and poor outcomes; the non-cardioembolic class displayed an intermediate profile, largely attributable to the higher prevalence of mild strokes.

The distributions of stroke severity at admission and discharge outcomes across latent classes, as shown in Supplementary Table 4 and mirrored in the bar plot of Supplementary Fig. 3, highlight the contrasting recovery trajectories across phenotypes.

On logistic regression analysis, no independent associations were found between the three LCA classes and poor functional outcome at discharge; although a non-significant trend towards poor functional recovery was observed for patients in the cardioembolic class compared to ESUS class (OR 1.32; 95% CI 0.92–1.91) (Fig. 4 and Supplementary Table 5).

### Sub latent class analysis among ESUS patients

To investigate residual heterogeneity within the ESUS class, we performed a LCA restricted to these patients. This analysis identified three clinically distinct subgroups:

Class A (middle-age dysmetabolic) comprised 70 patients (median age 60 years, IQR 53–66) with male predominance (91.4% male), and a high prevalence of hypertension (69%), dyslipidaemia (53%), diabetes (19%), and obesity (16%). Stroke severity was mild in 48.6%, moderate in 34.3%, and severe in 17.1% of cases. Pre-hospital antiplatelet therapy was recorded in 25.7%.

Class B (low cardiovascular young) included 33 patients (median age 45 years, IQR 37–51), mainly female (63.6%), with no hypertension, diabetes, or obesity; dyslipidaemia was reported in 27.3%. Stroke severity was mild in 39.4%, moderate in 36.4%, and severe in 24.2%. Pre-hospital antiplatelet and anticoagulant use were 6.1% and 0%, respectively.

Class C (cardiovascular elderly) consisted of 112 patients (median age 79 years, IQR 75–83), mainly females, with a high prevalence of hypertension (83.9%), diabetes (7.1%), dyslipidaemia (24.1%), and obesity (4.5%). Stroke severity was mild in 28.6%, moderate in 46.4%, and severe in 25.0%. Pre-hospital antiplatelet and anticoagulant therapy were reported in 32.1% and 4.5%, respectively (Table 4).

**Table 4:** Clinical characteristics across latent classes in ESUS stroke patients

Characteristic	Class A *N* = 70^*1*^	Class B *N* = 33^*1*^	Class C *N* = 112^*1*^	p-value^*2*^
Age, years	60 (53, 66)	45 (37, 51)	79 (75, 83)	< 0.001
Female	6 (8.6%)	21 (64%)	70 (63%)	< 0.001
Hypertension	48 (69%)	0 (0%)	94 (84%)	< 0.001
Diabetes	13 (19%)	0 (0%)	8 (7.1%)	0.005
Dyslipidaemia	37 (53%)	9 (27%)	27 (24%)	< 0.001
Obesity	11 (16%)	0 (0%)	5 (4.5%)	0.005
NIH baseline				0.10
Mild (0–4)	34 (49%)	13 (39%)	32 (29%)	
Moderate (5–15)	24 (34%)	12 (36%)	52 (46%)	
Severe (≥ 16)	12 (17%)	8 (24%)	28 (25%)	
Antiplatelet pre-hospital	18 (26%)	2 (6.1%)	36 (32%)	0.011
Anticoagulant therapy pre-hospital	3 (4.3%)	0 (0%)	5 (4.5%)	0.7

^*1*^Median (Q1, Q3); n (%)

^*2*^Kruskal–Wallis rank sum test; Pearson’s Chi-squared test; Fisher’s exact test

The in-hospital composite outcome occurred in 14.9% of the middle-age dysmetabolic class (Class A), 3.2% of the low cardiovascular young class (Class B), and 13.9% of the cardiovascular elderly class (Class C). In a logistic regression models, both middle-age dysmetabolic class and cardiovascular elderly class were associated with a higher odd of composite outcome compared to low cardiovascular young (OR 1.52, 95% CI 1.18–2.83; OR 1.56, 95% CI 1.21–2.77) (Supplementary Table 6).

## Discussion

In this study, our principal findings are as follows: i) IS clustered into three distinct clinical phenotypes, each associated with different patterns of acute management. ii) These phenotypes demonstrated divergent prognostic trajectories, with the ESUS class exhibiting a more favourable early prognosis compared to the cardioembolic class, and a trend towards worse clinical outcomes relative to the non-cardioembolic class. iii) Within the ESUS group, three additional sub-phenotypes emerged: the first comprised middle-aged males with features of dysmetabolism; the second included young women with a low cardiovascular risk profile; and the third consisted of elderly women with a high burden of cardiovascular comorbidities.

Our identification of distinct IS phenotypes highlights the heterogeneity of pathophysiology and clinical course. Conventional frameworks such as TOAST do not fully capture the interplay of demographic, vascular, and systemic factors. By initially applying a data-driven latent class approach based solely on clinical characteristics, we identified three distinct clinical profiles. The subsequent incorporation of stroke aetiology into the model yielded a refined yet largely comparable class structure, supporting the robustness of the initial classification for the cardioembolic and non-cardioembolic classes, while providing additional granularity in the distribution of patients within the ESUS class.

This stepwise modelling strategy reinforces the value of LCA for phenotype discovery, enabling classification schemes that go beyond traditional taxonomies and inform risk stratification and management in a clinically relevant manner.

In the full model, the first class reflected a non-cardioembolic phenotype, encompassing patients with a high prevalence of traditional cardiovascular risk factors. These individuals were predominantly male, exhibited mild-to-moderate neurological impairment, and had diagnoses largely consistent with large artery or small vessel disease. This cluster aligns with the classical construct of atherosclerotic stroke [19] and despite the high cardiovascular risk burden, the relative absence of cardioembolic sources may contribute to a more predictable clinical course.

The second phenotype was fully compliant with cardioembolic profile, characterised by older age, predominantly female, a high prevalence of cardiovascular burden [20], and AF as discriminant feature. These patients presented with more severe neurological deficits and more frequently received anticoagulation prior to stroke. This profile reflects the prototypical AF-related stroke, where the intrinsic thromboembolic predisposition, associated with advanced age and multimorbidity converge to produce more severe outcomes [21]. The clustering of clinical features in this phenotype echoes previous descriptions of cardioembolic stroke in elderly, often multimorbidity patients, and highlights the need for tailored management pathways [22, 23].

In contrast, the third phenotype captured a group of younger, predominantly male patients, with no AF and a relatively lower cardiovascular burden. However, cardiovascular risk factors such as hypertension and dyslipidaemia were still frequently observed, suggesting a potential role for metabolic or subclinical vascular contributors. Although neurological presentations were often mild, the class displayed a surprisingly broad clinical spectrum, with a non-negligible proportion of severe strokes. This internal heterogeneity, together with the absence of an identifiable embolic source, points towards a complex and multifactorial substrate. Notably, the clinical profile observed in this group aligns closely with recent findings from a prospective ESUS cohort, in which patients shared similar demographic and cardiovascular features [24].

Management approaches varied significantly across the clinical phenotypes. Patients within the non-cardioembolic class were more frequently treated with DAPT, consistent with current recommendations for minor or moderate non-cardioembolic strokes, particularly in the presence of presumed large artery disease [25, 26]. By contrast, IVT was used less frequently in the cardioembolic class than in the ESUS group, despite a more severe neurological presentation. This difference may partly reflect clinical decision-making in older age, frailty, and prior anticoagulation in the cardioembolic class likely contributed to therapeutic hesitation regarding reperfusion [27, 28]. In contrast, patients in the ESUS class were significantly more likely to receive IVT than those in the cardioembolic class. This appears driven by their younger age, fewer comorbidities, and the absence of anticoagulant therapy, factors that confer a lower perceived bleeding risk and fewer formal contraindications to treatment. Overall, these differences likely reflect real-world clinical decision-making across phenotypes, shaped by stroke subtype, baseline severity, comorbidity burden, and treatment eligibility.

Patients in the ESUS phenotype experienced fewer early adverse events than those in the cardioembolic class, a divergence that likely reflects the older age, greater frailty, higher AF burden, and more severe baseline stroke presentation in the cardioembolic group. These findings are consistent with established evidence indicating that AF-related strokes are associated with worse early outcomes and more frequent systemic complications than non-AF strokes [29]. Notably, our results also align with recent data reporting better early prognosis in ESUS patients compared to those with cardioembolic strokes [30].

Despite receiving IVT more frequently, patients in the ESUS class experienced notably lower rates of HT compared to those with cardioembolic stroke. A similar pattern was observed for sICH, which also occurred less frequently in ESUS, reinforcing the notion of a lower haemorrhagic risk profile in this subgroup. Patients with ESUS were generally younger, had fewer vascular comorbidities, and exhibited lower baseline stroke severity, all known protective features against haemorrhagic complications [31]. Conversely, AF-related strokes have been consistently associated with more extensive ischaemic damage, lack of collateral flow, and fragile microvascular integrity. Pooled analyses from the EPITHET and DEFUSE studies highlighted that AF strokes are prone to larger infarct growth and higher rates of parenchymal hematoma, even after adjusting for confounders such as age, NIHSS, and thrombolysis use [32, 33]. Thus, the lower HT and sICH rates observed in ESUS likely reflect a distinct pathophysiologic substrate, translating into a more favourable haemorrhagic risk profile and supporting a more confident use of reperfusion therapies compared to the greater caution warranted in AF-related strokes.

Functional outcome distributions differed descriptively across latent classes, with a more favourable recovery in ESUS than in the cardioembolic class. However, logistic regression did not show a significant association between latent class membership and functional recovery, suggesting that these differences were mainly driven by baseline stroke severity. This observation aligns with recent data comparing ESUS and cardioembolic strokes, where differences in mRS distributions were largely mediated by age and stroke severity than intrinsic differences in pathophysiology [34–36].

These findings challenge the conventional notion that ESUS represents merely latent AF-related events. This interpretation is supported by secondary prevention trials—most notably NAVIGATE ESUS and RE-SPECT ESUS—which failed to demonstrate a benefit of DOACs over aspirin in unselected ESUS populations [11, 12]. These neutral results reinforce the idea that ESUS is not a homogeneous entity, but rather a collection of non-lacunar embolic strokes lacking overt sources, often unrelated to AF.

The emerging clinical challenge, therefore, is to identify meaningful subgroups within the ESUS population that may benefit from tailored diagnostic and therapeutic strategies. In line with this, our exploratory sensitivity analysis sought to delineate phenotypic heterogeneity within ESUS strokes.

The ‘Middle-age Dysmetabolic’ class, characterised by middle-aged males with a high prevalence of cardiovascular risk factors and frequent pre-hospital use of antiplatelet therapy, may suggest an underlying atherothrombotic process—potentially involving stable, non-stenotic atherosclerotic plaques in the carotid arteries or aortic arch—which may reflect the non-cardioembolic class identified in the full LCA model [37]. In this group, vascular assessment focussed on the detection of atherosclerotic disease—including non-stenotic lesions—should be prioritised, even when standard imaging appears unremarkable.

Lastly, the ‘Cardiovascular Elderly’ class comprised older patients, predominantly women, with high prevalence of cardiovascular risk factors. Although none had a prior diagnosis of AF, their overall clinical profile closely mirrored that of the established cardioembolic class identified in the full LCA model. This suggests that subclinical AF, occult paroxysmal AF or other arrhythmogenic mechanisms may have contributed to the index event. Notably, this class resembles the subset of ESUS patients described in previous studies, in whom prolonged cardiac monitoring has frequently led to post-stroke detection of AF [38–40]. In light of the sub-LCA, we believe that these patients should be prioritised for extended rhythm monitoring to enhance AF detection and guide secondary prevention strategies [41].

By contrast, the ‘Low cardiovascular Young’ class comprised younger individuals, predominantly female, with no cardiovascular risk factors and a low risk of early adverse events. The pathophysiological substrate in this group remains unclear. It is plausible that this phenotype includes under-recognised embolic mechanisms such as paradoxical embolism, occult malignancy, autoimmune diseases or thrombotic diathesis [37]. As highlighted in recent consensus guidance, ESUS patients with no evident cause may benefit from broader diagnostic algorithms that consider cumulative thromboembolic risk beyond single aetiologic labels [37].

Our findings underscore the clinical and pathophysiological heterogeneity of ESUS population. This phenotypic granularity may help explain the neutral results of empiric anticoagulation trials in unselected ESUS populations and supports the urgent need for individualised diagnostic and therapeutic algorithms. These findings should be validated in larger, prospective cohorts to determine whether phenotype-driven strategies—such as targeted rhythm monitoring, tailored antithrombotic regimens, or advanced imaging protocols—can enhance diagnostic accuracy and ultimately lead to improved clinical outcomes in this heterogeneous and challenging stroke subgroup.

### Limitations

This study has some limitations. First, it was conducted at a single centre, which may have affected the generalisability of the findings. Second, due to its retrospective design, causal inferences cannot be drawn and residual confounding cannot be excluded. Although LCA was used to capture the joint distribution of multiple clinical variables, the observed association between LCA classes and clinical events should be regarded as exploratory and descriptive, as differences in outcomes may partly reflect remaining imbalances in prognostic features. Third, the lack of post-discharge follow-up restricted the analysis to in-hospital outcomes, limiting insight into medium- or long-term prognostic implications. In addition, the exact timing of in-hospital events was not systematically recorded, precluding formal time-to-event and competing-risk analyses. Fourth, the ascertainment of cardiac complications may have been incomplete, particularly in patients with severe neurological impairment or reduced consciousness, potentially leading to underestimation of their true incidence. Fifth, patients with missing data were excluded from the LCA, which may have introduced selection bias and reduced the representativeness of the analytical cohort. Although included and excluded patients were compared, residual differences between these groups may limit the external validity of the phenotypic classification. Sixth, the secondary LCA restricted to ESUS patients included a relatively small sample size and should therefore be considered exploratory and hypothesis-generating. These ESUS sub-phenotypes require confirmation in larger independent cohorts. Seventh, the long observational period (2005–2019) may have introduced heterogeneity in clinical management, including treatment allocation according to stroke subtype, severity, comorbidity burden, and eligibility criteria. Finally, our population was essentially white European, and additional studies are warranted in different ethnicities and healthcare settings, given the reported racial differences in stroke [42].

## Conclusion

Acute IS represents a clinically heterogeneous entity; ESUS phenotype is distinct from cardioembolic strokes in both management patterns and short-term prognosis. The identification of three ESUS sub-phenotypes underscores the need for more personalised and holistic approaches to diagnosis and secondary prevention.

## Supplementary Information

Below is the link to the electronic supplementary material.

Supplementary file1 (DOCX 281 KB)

## Data Availability

The datasets analysed during the current study are available from the corresponding author on reasonable request.
