# Supplementary material for: Clinical phenotypes of ischaemic stroke patients and early cardiovascular complications: a latent class analysis from the Perugia Stroke Registry
Source: Intern Emerg Med. 2026 Jun 10;21(7):2521–33. doi: 10.1007/s11739-026-04420-z (PMC13694437; doi:10.1007/s11739-026-04420-z)
Supplement: Supplementary file 1 [file 11739_2026_4420_MOESM1_ESM.docx]

**Supplementary methods**

Andrea Galeazzo Rigutini, Valeria Caso, Tommaso Bucci, Maria Giulia Mosconi,

Virginia Cancelloni, Andrea Alberti, Michele Venti, Ilaria Leone de Magistris, Alessandro Bufi, Chiara Urbini, Allegra Gaboni, Michele Marvardi, Carmen Rapuano, Wenhui Li,

Cecilia Becattini, Gregory Y. H. Lip and Maurizio Paciaroni

**Supplementary Table 1. Comparison of baseline clinical characteristics between patients included in the latent class analysis and those excluded due to missing data**

| Characteristic | Excluded n=598 | Included n=1,533 | p-value2 |
| --- | --- | --- | --- |
| Age, years | 74 ± 13 | 73 ± 12 | 0.11 |
| Female | 257 (43%) | 664 (43%) | 0.02 |
| Hypertension | 466 (78%) | 1197 (78%) | 0.91 |
| Atrial Fibrillation | 139 (23%) | 534 (35%) | <0.001 |
| Diabetes | 152 (25%) | 405 (26%) | 0.68 |
| Dyslipidaemia | 222 (37%) | 526 (34%) | 0.31 |
| Obesity | 60 (10%) | 142 (9.3%) | 0.65 |
| NIH baseline | 9 ± 7 | 9 ± 7 | 0.87 |
| Antiplatelet pre-hospital | 247 (41%) | 575 (38%) | 0.25 |
| Anticoagulant therapy pre hospital | 70 (12%) | 167 (11%) | 0.44 |

**Supplementary Table 2. Clinical Characteristics Across Latent Classes in Ischaemic Stroke Patients (first model)**

| Characteristic | Class 1 N = 763*^1^* | Class 2 N = 479*^1^* | Class 3 N = 291*^1^* | **p-value***^2^* |
| --- | --- | --- | --- | --- |
| Age, years | 75 (69, 80) | 82 (77, 86) | 56 (49, 63) | <0.001 |
| Female | 268 (35%) | 326 (68%) | 70 (24%) | <0.001 |
| Hypertension | 692 (91%) | 359 (75%) | 146 (50%) | <0.001 |
| Atrial Fibrillation | 107 (14%) | 374 (78%) | 53 (18%) | <0.001 |
| Diabetes | 291 (38%) | 71 (15%) | 43 (15%) | <0.001 |
| Dyslipidaemia | 317 (42%) | 78 (16%) | 131 (45%) | <0.001 |
| Obesity | 74 (9.7%) | 40 (8.4%) | 28 (9.6%) | 0.7 |
| NIH baseline |  |  |  | <0.001 |
| Mild (0-4) | 311 (41%) | 52 (11%) | 122 (42%) |  |
| Moderate (5-15) | 395 (52%) | 227 (47%) | 131 (45%) |  |
| Severe (≥16) | 57 (7.5%) | 200 (42%) | 38 (13%) |  |
| Antiplatelet pre-hospital | 433 (57%) | 130 (27%) | 12 (4.1%) | <0.001 |
| Anticoagulant therapy pre hospital | 0 (0%) | 148 (31%) | 19 (6.5%) | <0.001 |
| *^1^* Median (Q1, Q3); n (%) | | | | |
| *^2^* Kruskal-Wallis rank sum test; Pearson’s Chi-squared test | | | | |

**Supplementary Table 3. Incidence and multivariable logistic regression of symptomatic intracranial haemorrhage across stroke classes among patients with haemorrhagic transformation.**

| Classes | sICH n (%) | p-value* | OR (95% CI) | p-value* |
| --- | --- | --- | --- | --- |
| ESUS | 2 (11.1%) | 0.211 | Reference | – |
| Non-cardioembolic | 8 (30.8%) |  | 3.56 (0.75–25.92) | 0.140 |
| Cardioembolic | 12 (17.4%) |  | 1.68 (0.40–11.54) | 0.520 |

**Supplementary Table 4. Distribution of Stroke Severity at Admission and Functional Outcome at Discharge According to Latent Class**

| Latent Class | Mild Good (%) | Mild Poor (%) | Moderate Good (%) | Moderate Poor (%) | Severe Good (%) | Severe Poor (%) |
| --- | --- | --- | --- | --- | --- | --- |
| Non-cardioembolic | 219 (80.2%) | 54 (19.8%) | 158 (40.6%) | 230 (59.3%) | 2 (2.5%) | 78 (97.5%) |
| Cardioembolic | 101 (84.9%) | 18 (15.1%) | 84 (32.4%) | 175 (67.6%) | 6 (3.7%) | 156 (96.3%) |
| ESUS | 72 (92.3%) | 6 (7.7%) | 39 (46.4%) | 45 (53.6%) | 4 (8.5%) | 43 (91.5%) |

**Supplementary Table 5. Univariable Logistic Regression Assessing the Association Between Latent Classes and Poor Functional Recovery at Discharge**

|  | **Univariable analysis**  **OR (95% CI)** | **p-value***^2^* |
| --- | --- | --- |
| **Poor Functional Recovery** | | |
| Non-cardioembolic class | 0.80 (0.56–1.15) | 0.23 |
| Cardioembolic class | 1.32 (0.92–1.91) | 0.13 |
| ESUS class | Reference | |

**Supplementary Table 6. In-Hospital Incidence rate and univariable logistic regression about composite outcome according to latent classes in ESUS sensitivity**

|  | **Number of events (within class %)** | **Univariable analysis**  **OR (95% CI)** | **p-value***^2^* |
| --- | --- | --- | --- |
| **Composite outcome** | | | |
| Middle-age Dysmetabolic | 10 (14.9%) | 1.52 (1.18–2.83) | 0.059 |
| Cardiovascular Elderly | 15 (3.2 %) | 1.56 (1.21–2.77) | 0.053 |
| Low cardiovascular Young | 1 (13.9%) | Reference | |

**Supplementary Figure 1. Flow chart study**

**Supplementary Figure 2. Comparison of Median Posterior Probability Distribution Between LCA reduced model and full model**

|  | LCA Reduced Model | Full Model |
| --- | --- | --- |
| Minimum | 0.355 | 0.988 |
| 1st Quartile | 0.706 | 1 |
| Median | 0.866 | 1 |
| Mean | 0.822 | 0.9997 |
| 3rd Quartile | 0.964 | 1 |
| Maximum | 1 | 1 |

**Supplementary Figure 3. Relationship Between Stroke Severity, Latent Class Profiles, and Functional Recovery at Hospital Discharge**

**Supplementary Methods 1**

*Study Design and Population*

This study is based on data from the Perugia Hospital-Based Stroke Registry (PSR), a retrospective, observational, hospital-based registry that includes consecutive patients admitted to the Stroke Unit of the “Santa Maria della Misericordia University Hospital” (Perugia, Italy). Inclusion in the study did not influence patient management at any stage of in-hospital care. The study period spanned from March 12, 2005 to September 20, 2019. During this period, the Stroke Unit admitted an average of ~190 patients per year. All patients were evaluated by a board-certified neurologist. Patients were eligible if they were admitted with clinical signs of a focal disturbance of cerebral function and received a radiologically confirmed diagnosis of either IS or haemorrhagic stroke based on non-contrast computed tomography (CT) or magnetic resonance imaging (MRI) performed on admission. Patients with subarachnoid haemorrhages, venous sinus occlusion and stroke mimics were excluded. Every patient underwent brain CT scan without contrast in the acute phase and this was repeated 3–7 days from stroke onset. CT scan with contrast medium was performed only in selected patients. Additional tests, performed in all patients, included: Echo-Power Doppler ultrasonography with frequency spectral analysis (common, external, and internal carotid arteries; vertebral arteries at the intra- vertebral level), 12-lead electrocardiography, standard blood and urine tests including venous haematocrit and fasting blood glucose and cholesterol concentration. Selected patients underwent cerebral magnetic resonance imaging, angio-MRI, cerebral angiography (Seldinger technique), two-dimensional echocardiography, and 24-h ECG (Holter) monitoring.

*Data Collection*

The following data were collected: age, sex, hypertension (blood pressure of > 160/90 mmHg at least twice before stroke or already under treatment with antihypertensive drugs), diabetes mellitus (diagnosed previously), cigarette smoking (>10 cigarettes per day or cessation <5 years earlier), family history (stroke and/or heart disease), alcohol abuse (>300 g/week) , previous transient ischemic attacks, dyslipidaemia, obesity, sleep apnoea, depression, atrial fibrillation (AF) , peripheral artery disease, admission and discharge date of hospitalization, pre-hospital therapies, in-hospital therapies including dual antiplatelet therapy (DAPT), intravenous thrombolysis (IVT), and endovascular therapy (EVT), in-hospital complications (all cause of death, acute myocardial infarction , heart failure, pulmonary embolism, deep vein thrombosis and laboratory tests. An experienced neuroradiologist determined stroke location, blinded to the clinical subtype of stroke. Stroke aetiology was classified by an experienced neurologist using the TOAST criteria (3), which categorise IS as large artery atherosclerosis, cardioembolic, small vessel occlusion, other determined aetiology, undetermined aetiology, or multiple possible causes. Haemorrhagic transformation (HT) was systematically assessed using follow-up neuroimaging during hospitalisation. The presence of HT was defined according to the European Cooperative Acute Stroke Study (ECASS) criteria (16), including any haemorrhagic infarction or parenchymal haemorrhage. Symptomatic intracerebral haemorrhage (sICH) was defined according to the ECASS II/III criteria, as the presence of a parenchymal haemorrhage on neuroimaging associated with a clinical deterioration of ≥4 points on the NIHSS or death (17). Stroke severity was assessed using the National Institutes of Health Stroke Scale (NIHSS) at admission and discharge. Functional status was assessed using the modified Rankin Scale (mRS) at hospital admission and at discharge.

**Supplementary Methods 2**

**Latent class analysis**

The term ‘latent’ refers to the fact that this method identifies classes that cannot be directly observed by the investigators. LCA calculates the probability of each participant belonging to each latent class based on observed characteristics.

To define the latent classes, we constructed two models. The first model included the following clinical variables: age, sex, hypertension, diabetes mellitus, dyslipidaemia, obesity, AF, and stroke severity at admission, assessed by the NIHSS. The second model (full model) included all the variables from the first model, with the addition of stroke aetiology. Stroke aetiology was classified according to the TOAST criteria (3), considering only the following subtypes: large artery atherosclerosis, small vessel occlusion, cardioembolic stroke, and ESUS.

This two-model approach was used to evaluate the consistency of clinical profiles in the absence of stroke aetiological information and to compare the accuracy of each model in assigning patients to clusters.

In particular, we aimed to explore whether ESUS patients could be phenotypically distinguished from those with cardioembolic or non-cardioembolic strokes—two well-established and mechanistically defined subtypes. To further characterise the heterogeneity within the ESUS population, we conducted a sensitivity analysis restricted to patients classified as ESUS class. A separate LCA was performed in this subgroup, with the goal of identifying clinically meaningful ESUS sub-phenotypes based on demographic, vascular, and stroke severity variables. This model was designed to refine the phenotypic understanding of ESUS beyond conventional classification schemes.

The optimal number of classes for both models was determined by minimising the Bayesian Information Criterion and the consistent Akaike Information Criterion, supported by clinical judgement. Each patient was assigned to the class for which they had the highest posterior probability of membership.

Model performance and classification precision were evaluated by comparing the distribution of posterior probabilities of class membership between the reduced and full models.

**Supplementary Results 1**

**Classes of patients: LCA full model**

Class 1 included 761 patients and was characterised by an intermediate age profile (median 74 years, IQR 66–81) and a predominance of male sex (64%). This group encompassed exclusively non-cardioembolic strokes, with 61% of patients classified as atherothrombotic and the remaining 39% as small vessel disease. Hypertension emerging as the predominant comorbidity (84%). A metabolic profile characterized by diabetes (35%) and dyslipidaemia (41%) was also frequently observed, further contributing to the overall vascular risk burden. AF was virtually absent (0.3%), and the use of pre-hospital oral anticoagulation was limited to 2.8%, whereas antiplatelet therapy was reported in 40% of patients. This class exhibited the most favourable stroke severity profile, with the highest rates of mild (42%) and moderate (47%) presentations, and only 11% of patients showing severe neurological impairment (NIHSS ≥16).

Class 2 comprised 557 patients and represented the oldest group (median 78 years, IQR 71–84), with a higher proportion of women (53%). All patients in this class were classified as cardioembolic stroke, and AF was almost universally present (96%). Compared with Class 1, this group had a slightly lower prevalence of hypertension (75%) and markedly lower rates of diabetes (22%) and dyslipidaemia (25%). Pre-stroke anticoagulant use was substantially more common (25%), reflecting the arrhythmic substrate, while antiplatelet therapy was used in 39% of cases. This class exhibited the most severe neurological profile overall, with the highest proportion of patients classified as severe (30%) or moderate (56%) and the lowest prevalence of mild strokes (14%), indicating the most unfavourable stroke presentation among all classes.

Class 3 included 215 patients and was the youngest across the cohort (median age 70 years, IQR 55–79), with a slight male predominance (55%). This class was characterized by a stroke phenotype consistent with embolic stroke of undetermined source (ESUS), in the absence of atrial fibrillation, large artery atherosclerosis, or small vessel disease. Patients in this group had the lowest prevalence of hypertension (66%) and diabetes (9.8%) among all classes, while dyslipidaemia remained relatively common (34%). Pre-stroke use of antithrombotic therapy was the lowest across the cohort, with only 26% of patients on antiplatelets and 3.7% on anticoagulants. Stroke severity was frequently mild (37%) or moderate (41%), yet a notable proportion (22%) presented with severe neurological deficits, exceeding the rate observed in Class 1.

Overall, the three latent classes delineated distinct clinical phenotypes: Class 1 was characterized by a high burden of vascular risk factors; Class 2 reflected a cardioembolic profile, driven by AF and severe stroke presentation; and Class 3 comprised a younger, less comorbid population with ESUS.
